# Research on Precision Blanking Process Design of Micro Gear Based on Piezoelectric Actuator

**DOI:** 10.3390/mi12020200

**Published:** 2021-02-15

**Authors:** Changjun Hu, Yunyang Shi, Fangfang Liu

**Affiliations:** School of Mechanical and Electrical Engineering, Suqian College, Suqian 223800, China; 18121@squ.edu.cn (Y.S.); 18114@squ.edu.cn (F.L.)

**Keywords:** piezoelectric ceramics, control method, actuator, precision micro forming

## Abstract

In order to process micro scale parts more conveniently, especially the micro parts with complex shape, a new micro blanking equipment based on piezoelectric ceramic driving is proposed in this paper. Compared with other large precision machining equipment, the equipment cost has been greatly reduced. Using displacement sensor to detect the change of output displacement and feedback control piezoelectric actuator to control the change of relevant parameters, the control precision is high. The micro gear parts with diameter less than 2 mm are obtained through the blanking experiment on the experimental equipment. From the relationship between the obtained time and the punch output force, output displacement and die adjustment, it can be seen that the designed equipment has good processing performance and can complete the blanking forming of micro parts well.

## 1. Introduction

The miniaturization of industrial equipment, especially medical equipment and electronic equipment, is to meet the requirements of the development of modern medical science and precision instruments, so the components of these equipment must be more miniaturized and high-precision. For these micro scale parts, different from traditional size parts, special processing methods must be used. For example, laser dynamic flexible punching process, electrical-field activated sintering, metal powder compaction in elastic dies, and micro wire electrical discharge machining are the common processing methods studied at present [1,2,3,4]. For gears of traditional size, we have various mature processing technologies, which can meet the technological requirements of various traditional equipment for gears. However, the micro gear cannot be realized by the traditional cutting technology. The thickness of the gear teeth is very thin. When the traditional technology is used for processing, the gear teeth are easily broken. At present, some mold manufacturers in the mechanical industry can obtain the pinion by wire cutting, but the size of the gear they can process cannot be too small, because once the size of the gear is less than 1–2 mm, very thin electrode wire must be competent, which will greatly increase the cost [5,6,7,8,9,10]. Maher et al. provided a method for increasing the productivity of the wire-cut electrical discharge machine [11]. Chen et al. analyzed the geometrical inaccuracy in WEDM rough corner cutting [12]. Jianwen et al. studied micro gear fabrication in laser dynamic flexible punching [13]. Horiuchi et al. put forward micro gear fabrication using optical projection lithography on copper-clad plastic substrates and electroplating of nickel [14]. Cannella, E.; Nielsen, E.K.; Stolfi, A. designed a tool system for lowering friction during the ejection of in-die sintered micro gears [15]. Garner, S.; Ruiz, E.; Strong, J.; Zavaliangos, A. studied mechanisms of crack formation in die compacted powders during unloading and ejection [16]. Akarachkin, S.A.; Ivashutenko, A.S.; Martyushev, N.V. studied activation of mass transfer processes at spark plasma sintering of zirconium dioxide [17]. Zhong et al. provided micro-stepped gear mold fabrication based on WEDM and thermal diffusion welding [18]. Álvarez et al. provided a large spiral bevel gear fabrication on universal 5-axis milling machines [19]. Yohei and Hirota studied precision stamping process of metal micro gears [20,21]. Peng-Zhi et al. studied dynamic linear modeling, identification and precise control of a walking piezo-actuated stage—ScienceDirect [22]. Wang et al. established a new hysteresis modeling and analysis feed forward control of piezoelectric actuators based on asymmetric Prandtl-Ishlinskii model [23]. Peng-Zhi et al. studied “Piezoelectric Actuated Phase Shifter Based on External Laser Interferometer: Design, Control and Experimental Validation” [24]. Bo et al. studied “5-axis double-flank CNC machining of spiral bevel gears via custom-shaped milling tools—Part I: Modeling and simulation” [25]. The equipment designed in this paper can get micro gears by means of punching, with a diameter of addendum circle less than 2 mm, and the gear is a kind of tooth with addendum and root trimming. In order to make the blanking surface of the gear more fine, when the working material becomes very thin, it is necessary to provide a very narrow gap evenly between the punch and the die along the contour of the part. Generally, according to the results of precision blanking, the gap is set to about 1% of the thickness of the workpiece.

## 2. Experimental Device Design

### 2.1. Micro Gear Parts

**Figure 1:**
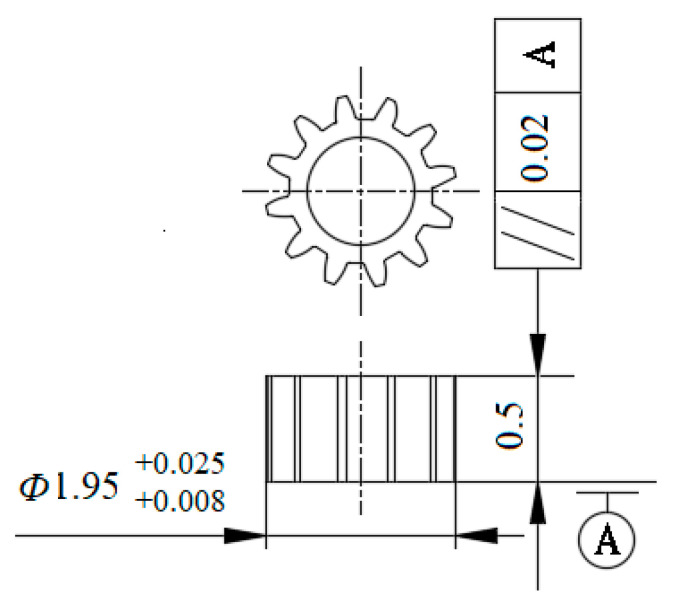
Part drawing of micro gear.

Figure 1 is the part drawing of the involute micro gear to be processed. The basic specification of the gear is that the reference circle diameter is 1.8 mm, the number of teeth is 12, and the module is 0.15 mm. The workpiece material is H62, the tooth thickness t is 235.5 μm, and the tooth width B is 0.5 mm. Table 1 shows the performance parameters of workpiece materials.

### 2.2. Structure of Experimental Device

**Figure 2:**
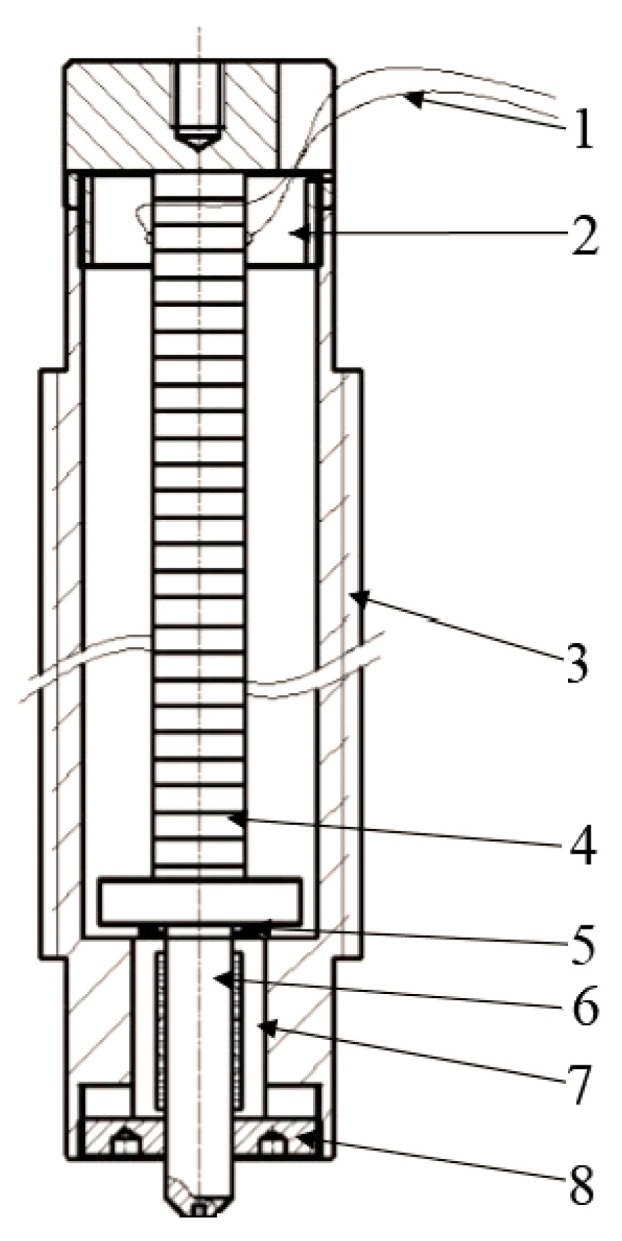
The internal structure of packaged piezoelectric ceramics. 1. Drive power line; 2. Fixed sea; 3. Package shell; 4. Laminated piezoelectric ceramics; 5. Disk spring; 6. Output shaft; 7. Linear bearing; 8. Locking cover.

**Figure 3:**
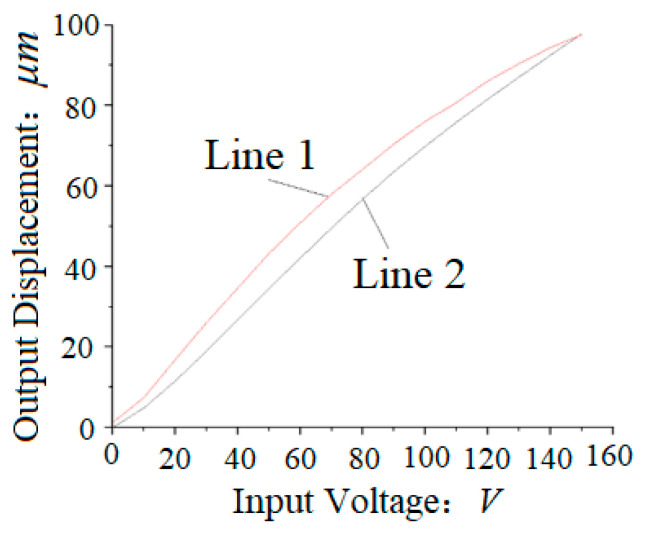
The open-loop voltage and displacement curve of piezoelectric ceramics. Line 1: Relationship between rising input voltage and measured output displacement; Line 2: Relationship between down input voltage and measured output displacement.

The deformation of single-layer ceramic is limited. In this project, the piezoelectric ceramic driver is designed by stacking multi-layer piezoelectric ceramic, as shown in Figure 2. The workpiece is punched and cut under the action of external voltage. In the piezoelectric ceramic stack structure, each piece of ceramic is embedded in the electrode to form a mechanical series connection and a circuit parallel connection. The relationship between the elongation of piezoelectric ceramic stack structure and input voltage is:

(1) $$\Delta l=n\xi u+nmu^{2}/t$$

where Δl is total elongation, *n* is the number of piezoelectric ceramics, ξ is piezoelectric coefficient, *u* is input voltage, *t* is the thickness of each piezoelectric ceramic, *n* = 27. Figure 3 shows the open-loop voltage and displacement curve of piezoelectric ceramics.

The relevant values of die root diameter and punch tip diameter are listed in Table 2:

### 2.3. Calculation of Precision Blanking Process Force

**Figure 4:**
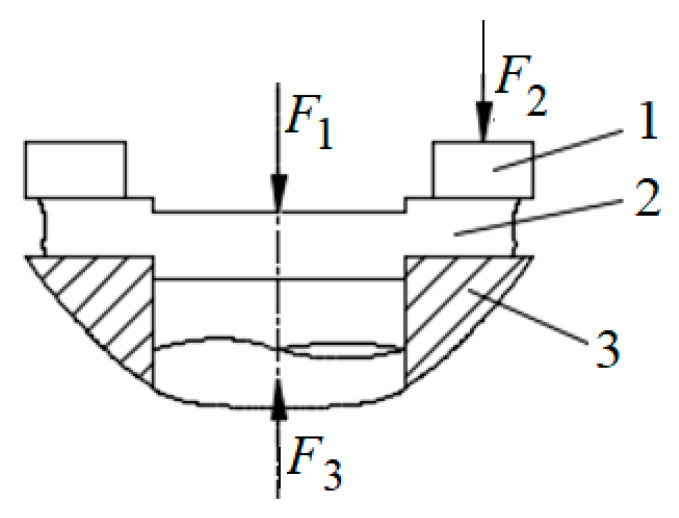
The stress during precision blanking. 1. Compaction block; 2. Workpiece; 3. Workbench.

The stress during precision blanking is shown in Figure 4. In the punching process, the workpiece receives the blank holder force *F*_2_, the punching force *F*_1_ and the counter pressure *F*_3_ at the same time.

#### 2.3.1. Calculation of the Punching Force

The blanking force *F*_1_ can be calculated by Formula (2):

(2) $$F_{1}=f_{1}l_{t}t\sigma_{b}$$

where $f_{1}$ is a coefficient that depends on the yield ratio σsσb of the material. Considering that the die gap is small and the material is in a three-dimensional compressive stress state during precision blanking, the deformation resistance is greater than that of general blanking, with $f_{1}=0.9$;

$l_{t}$ is the outline peripheral length;

*t* is the workpiece thickness;

$\sigma_{b}$ is the tensile strength of materials.

In order to get the contour length *l*, the involute length of one-side of gear teeth is calculated first [26], and its calculation formula is:

(3) $$l_{inv}=0.5r_{b}(tan^{2}\alpha_{a}-tan^{2}\alpha_{b})=0.5r_{b}tan^{2}\alpha_{a}$$

where $l_{inv}$ is the involute length;

$r_{b}$ is the radius of base circle;

$\alpha_{a}$ is the pressure angle of addendum circle;

$\alpha_{b}$ is the pressure angle of base circle.

The tooth thickness on different circles is calculated as Formula (4) [26,27]:

(4) $$\varphi_{d}=s/r-2(inv\alpha_{d}-inv\alpha )$$

where $\varphi_{d}$ is the central angle of tooth thickness on a circle with diameter *d*;

*s* is the tooth thickness of graduation circle;

*r* is the radius of reference circle;

$\alpha_{d}$ is the pressure angle of tooth thickness on a circle with diameter *d*;

$\alpha_{d}$ is the pressure angle of graduation circle.

The total length of contour circumference is obtained as Formula (5):

(5) $$l_{t}=2\times z\times l_{inv}+z\times s_{a}+(\pi d_{b}-z\times s_{b})$$

where *z* is teeth number of the gear;

$s_{a}$ is the tooth thickness on addendum circle;

$s_{b}$ is the tooth thickness on base circle;

$d_{b}$ is the diameter of base circle.

Comprehensive of Formulas (3)–(5), the calculation results of the total length of contour circumference are as follows:

$$l_{t}\approx 8.8228\mathrm{mm}$$

Substituting $l_{t}$, *t*, *f*_1_ and $\sigma_{b}$ into Formula (2), the value of blanking force *F*_1_ is:

$$F_{1}=f_{1}l_{t}t\sigma_{b}=0.9\times 8.8228\times 0.5\times 370\approx 1469N$$

#### 2.3.2. Calculation of the Blank Holder Force

The calculation method of blank holder force can be obtained from the empirical formula of Reference [27]:

$$F_{2}=f_{2}l_{t}2h\sigma_{b}=1.5\times 8.8228\times 2\times 0.3\times 370\approx 2938N$$

where *h* is v-shaped tooth height of blank holder.

#### 2.3.3. Calculation of the Counter Pressure

The value of the back pressure *F*_3_ will have an impact on the dimensional accuracy, flatness, collapse angle and the quality of the shear plane of the workpiece. Increasing the back pressure appropriately will improve the above indexes, but excessive back pressure will increase the punch load. Back pressure of fine blanking process can be calculated by Formula (6) [27]:

(6) $$F_{3}=pA$$

where *p* is unit back pressure, generally taken at 20~70 N/mm^2^, with *p* = 55 N/mm^2^; *A* is the end area of part; the calculation formula can be obtained from Reference [26].

(7) $$A=(0.5s/r+inv\alpha )(r_{a}^{2}-r_{b}^{2})-(inv\alpha_{a}r_{a}^{2}-inv\alpha_{b}r_{b}^{2})+2{r_{b}}^{2}(tan^{3}\alpha_{a}-tan^{3}\alpha_{b})/6$$

Substituting all data into Formula (7), we can obtain:

$$A=0.03265{\mathrm{mm}}^{2}$$

$$∴\;F_{3}=55\times 0.03265=1.796N$$

#### 2.3.4. Total Pressure Calculation

The total pressure required by the workpiece to finish the fine blanking process is the main basis for the design of stamping equipment. The workpiece should be blanked before fine blanking to ensure reliable blank pressing. The total pressure includes blanking force and back pressure, which can be calculated as follows:

$$F=F_{1}+F_{3}=1469+1.796=1470.8N$$

## 3. Simulation of Micro Gear Blanking

### 3.1. Simulation Model

**Figure 5:**
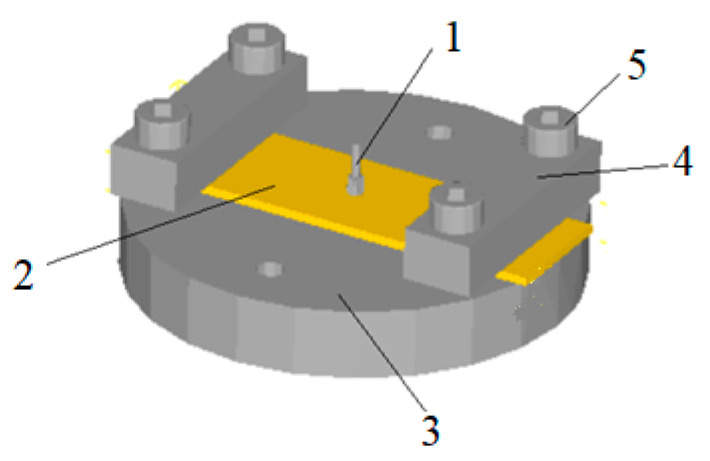
The simulation model of micro gear blanking processing. 1. Punch; 2. Workpiece; 3. Die; 4. Press plate; 5. Screw.

We used Solid Edge software to build 3D model and save it in STL format. Then, we imported STL file into DEFORM software and adjusted the position of each part to form the original model as shown in Figure 5. The blank is pressed on the upper surface of the gear die by a pressing block, and the punch is placed on the top surface of the blank.

**Figure 6:**
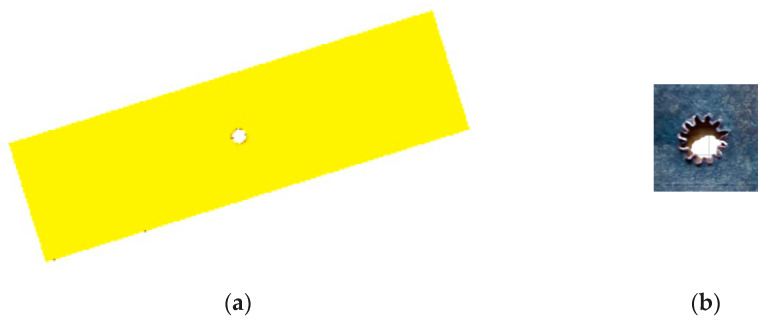
(**a**) The blank after the micro gear blanking simulation; (**b**) The blank after the micro gear blanking on the designed micro forming equipment.

Figure 6a is the blank after the micro gear blanking simulation, whose size is consistent with the actual size and Figure 6b is the blank after the micro gear blanking on the designed micro forming equipment, whose size and actual size ratio is 3:1. From Figure 6a, the simulation blanking is very close to the desired shape, so the model can get the simulation results more accurately, and can do further research on the blanking process of the designed equipment. From Figure 6b, we can see the teeth obtained on the equipment are with tip relief and bottom relief. D. Martínez Krahmer et al.’s research on the edge quality affected fatigue or tensile strength, whose results show that some changes on surface state appeared, but the effect on tensile strength was lower than 5% [28].

### 3.2. Analysis of Blanking Stress

**Figure 7:**
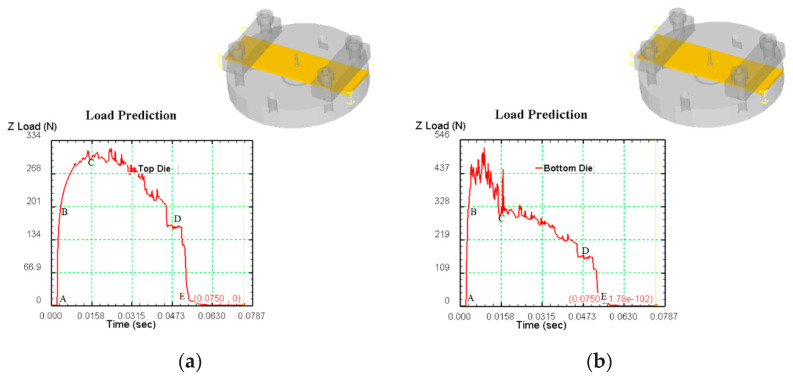
(**a**) The effective stress diagram of the punch in the simulation analysis of micro gear blanking; (**b**) The effective stress diagram of the die in the simulation analysis of micro gear blanking.

Figure 7a is the effective stress diagram of the punch in the simulation analysis of micro gear blanking. AB section is the stage of elastic deformation. At the beginning, the punch contacts with the blank and produces downward pressure. The blank is bent and deformed, and the blanking force increases approximately vertically in a short time until it reaches the yield strength of the blank. For yield deformation, such as BC section, the blanking force increases slowly and increases with the movement of the punch, reaching the yield strength at C point maximum. At this stage, the stress in the deformation zone of the blank exceeds its yield strength, the sheet metal is pressed into the die, plastic deformation occurs, and large stress occurs at the contact edge of the die, and then the stress value exceeds the shear strength, and the blank begins to crack. The blank starts to fracture in the CD section, the punch continues to move, the blanking force gradually decreases, the blank crack continues to extend until it is completely separated, the blanking process is completed, and then the blanking force decreases vertically.

Figure 7b is the effective stress diagram of the die in the simulation analysis of micro gear blanking. The blank of AB section is elastic deformation, and the blanking force increases approximately vertically in a short time until it reaches the yield strength of the blank. The blank produces yield deformation. At the beginning of BC section, the die stress transits from elastic deformation to plastic deformation step by step. At this stage, the die stress increases, and then decreases to C. At this point, the sheet metal is pressed into the die, and a large stress is generated at the contact edge of the die, and the stress value exceeds the shear strength. The blank begins to crack, the thickness of the blank joint decreases, the punch continues to move, the blanking force on the die gradually decreases, and the blank crack continues to extend and expand until it is completely separated. The blanking process ends, and then the blanking force in the DE section decreases vertically.

**Figure 8:**
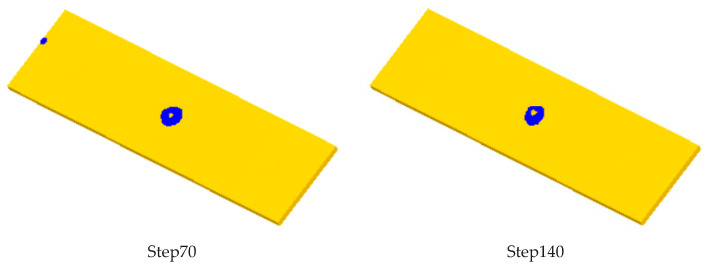

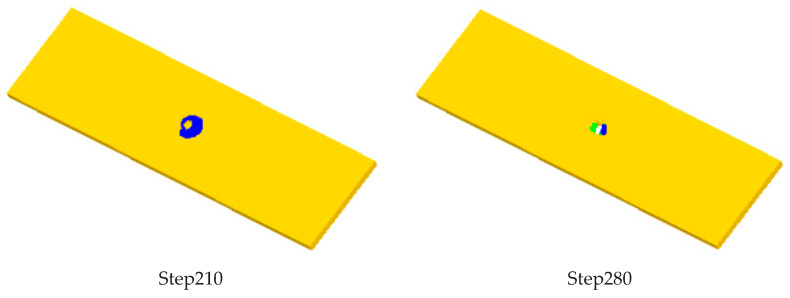
Stress effect of billet in different simulation steps.

There are three hundred steps in the whole simulation process. The effective strain of blank is shown in Figure 8 for every 70 steps. It can be seen that the model can obtain the ideal simulation results.

## 4. Experiment of Micro Gear Forming

### 4.1. Structure of Micro Gear Forming Device

**Figure 9:**
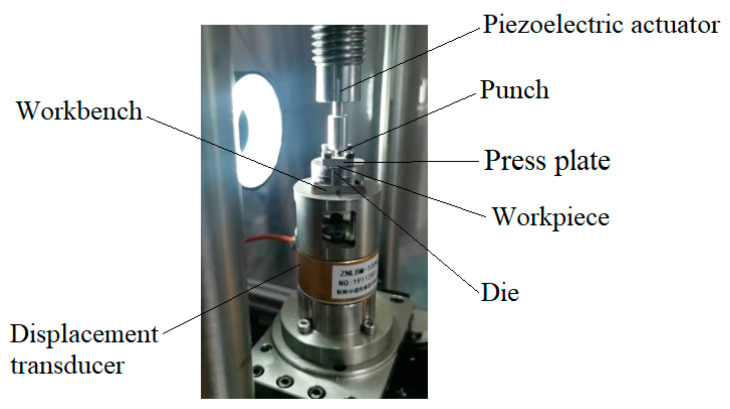
Stress effect of billet in different simulation steps.

Figure 9 shows the designed micro gear forming device, which uses the principle that piezoelectric ceramics can produce output displacement with the change of input voltage, and can produce larger output force to drive the punch to achieve stamping action. The blank is pressed on the upper surface of the die by the pressing block, and the die is fixed on the worktable. A displacement sensor is set under the worktable to obtain the impact displacement and feed it back to the controller. The punch is fixed on the upper bracket, and the punch and the die are strictly aligned during installation.

### 4.2. Change of Micro Gear Processing Parameters

**Figure 10:**
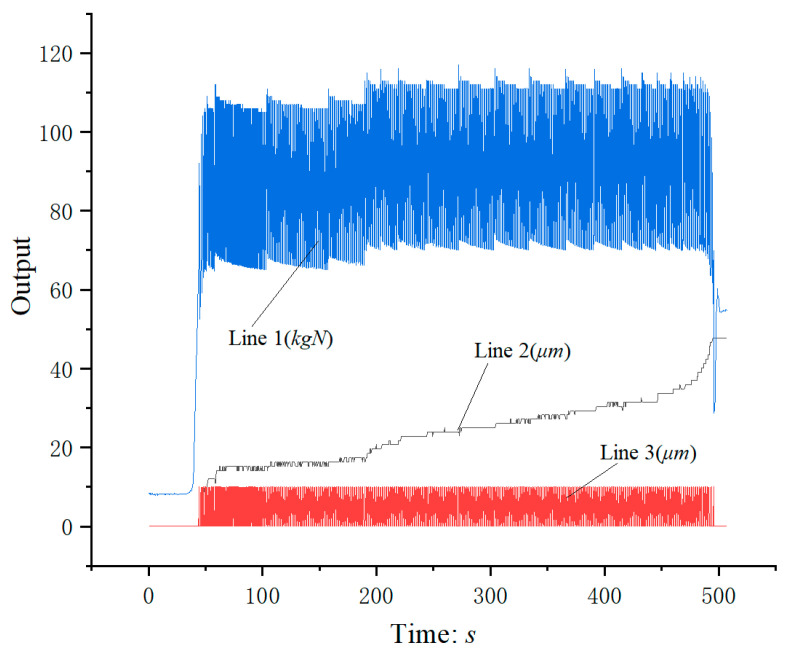
The relationship between output and time. Line 1: Diagram of punch impact force versus time; Line 2: Diagram of punch displacement versus time; Line 3: Diagram of die adjusting displacement with time.

Figure 10 shows the relationship between output and time. Line 1 is the diagram of punch impact force versus time; Line 2 shows the change between punch displacement and time; Line 3 is the diagram of die adjusting displacement with time. Micro gear parts can be easily obtained by reasonable parameter setting.

### 4.3. Micro Gear Parts Formed by Blanking on the Device

**Figure 11:**
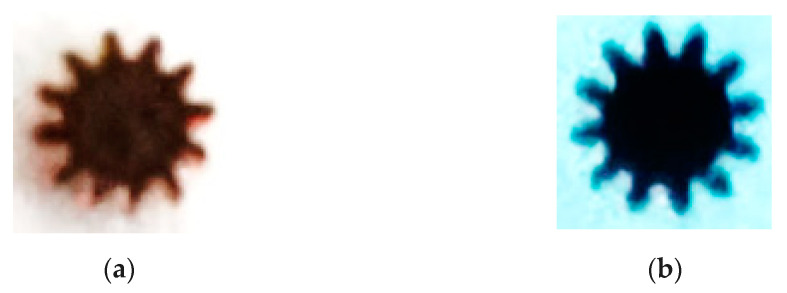
Micrograph of formed gear and die. (**a**) Micro gear parts formed by machining, (**b**) Partial screenshot of die gear teeth

Figure 11a shows the micro gear parts obtained, with module of 0.15 mm, number of teeth of 12 and tooth width of 0.5 mm. Figure 11b is a micrograph of the die. It can be seen from the figure that the profile of the formed gear is clear and there is no burr on the profile surface. The main reason is that during stamping, the blank of the part is fixed firmly and reliably, and the pressure stress produced by the punch is evenly distributed, the material is pressed into the die through several strokes of the punch, the surface of the part is extruded flat inside the die surface, with high dimensional accuracy and small surface roughness.

## 5. Discussion

A set of device for precision forming of micro parts based on piezoelectric ceramic driver is developed. (1) Through the displacement sensor detection and feedback, the position adjustment of the precision electric displacement table is realized, so as to complete the adjustment of the machining position of the parts. (2) The control system of the device is designed. By selecting the appropriate control parameters, the device can run stably and reliably. (3) The tooth profile of the micro gear is clear, and the microhardness meets the requirements of the gear, so the designed micro stamping system can form the micro parts with good quality.

## Figures and Tables

**Table 1:** Performance parameters of workpiece materials.

Item	Value
Material composition	Cu 60.5%~63.5%
Tensile strength (Mpa)	370
Modulus of elasticity (Gpa)	100
Vickers hardness (HV)	115
Coefficient of linear expansion (*106)	20.6
Fatigue limit (Mpa)	154
Yield strength (Mpa)	120

**Table 2:** Main parameters of blanking device.

Item	Value
Unilateral Gap CL (μm)	5
Addendum Circle Diameter of Punch dP_0_ (mm)	1.95
Root Circle Diameter of Die dD_0_ (mm)	1.85
Outer Diameter of Die dD (mm)	25.0
